# Acute Generalized Cutaneous Lupus Erythematosus Repeatedly Mistaken for Cellulitis

**DOI:** 10.7759/cureus.6946

**Published:** 2020-02-11

**Authors:** Nicole Fischer, Joseph Geffen, Mary Spring

**Affiliations:** 1 Internal Medicine, Nova Southeastern University, Dr. Kiran C. Patel College of Osteopathic Medicine, Ft. Lauderdale, USA; 2 Internal Medicine, Lake America Family Physicians, Clermont, USA; 3 Internal Medicine, Nova Southeastern University Dr. Kiran C. Patel College of Osteopathic Medicine, Ft. Lauderdale, USA

**Keywords:** acute cutaneous lupus erythematosus, acle, systemic lupus erythematosus, sle, cutaneous lupus erythematosus, lupus, cellulitis

## Abstract

Cutaneous lupus erythematosus (CLE) may occur in association with systemic lupus erythematosus (SLE) or independently of SLE. Among the various subtypes of CLE, acute cutaneous lupus erythematosus (ACLE) has the highest rate of occurrence in association with SLE rather than independently; thus, if a patient presents with ACLE, a workup for SLE should be performed if not already diagnosed. In this case, we present a 52-year-old female with a past medical history consistent with a diagnosis of SLE (including end-stage renal disease, antiphospholipid syndrome, and seizure disorder); however, the patient went undiagnosed for years. Thus, when she presented with an unusual presentation of ACLE, the diagnosis was initially overlooked.

## Introduction

Many patients with systemic lupus erythematosus (SLE) suffer from a variety of dermatological conditions. In fact, 80% of patients have skin lesions at some point in their disease, and up to one-fourth of patients have cutaneous findings at the point of diagnosis [1]. The cutaneous involvement seen in SLE is typically separated into “specific” and “non-specific” skin manifestations. The non-specific lesions include, but are not limited to, non-scarring alopecia, Raynaud’s phenomenon, vasculitic lesions, and oral ulcers. Using clinical and histological criteria, the specific lesions have been categorized into acute cutaneous lupus erythematosus (ACLE), subacute CLE, chronic CLE, and intermittent CLE [2-4].

ACLE has been further classified into localized or generalized forms. The localized form of ACLE presents as the classic malar rash that encompasses the facial cheeks and nasal bridge, sparing the nasolabial folds, and often parallels the severity and course of SLE, if present. It typically appears as an erythematous lesion, but is also known to have a fine layer of scale and edema at times as well. This rash often is brought on by sun exposure, which causes patients to misinterpret the lesion as a sunburn and not seek medical advice.

In contrast, the rarer, generalized form may affect areas above or below the neck, such as the lateral or extensor aspects of the arms, elbows, shoulders, knees, and truck in a photosensitive distribution. It may present in various manners, including an erythematous maculopapular rash, plaque-like lesions, a widespread area of congestive erythema, crusting, scaling lesions, or erosions [2]. Often times, the generalized form appears similar to a drug rash or infectious process, which may delay the diagnosis of ACLE. In both the localized and generalized subtypes, ACLE typically does not result in scarring of the skin after resolution of the lesion; however, dyspigmentation may occur [3].

## Case presentation

A 52-year-old female with end-stage renal disease (due to unknown cause) on peritoneal dialysis, chronic antiphospholipid syndrome (on warfarin), anemia of chronic disease, and seizure disorder presented urgently to her primary care physician with a small plaque-like abrasion on her right forearm. She was prescribed trimethoprim-sulfamethoxazole, which failed to improve the skin lesion, and her pain continued to worsen. She was, therefore, admitted to the hospital for intravenous (IV) antibiotics for presumed cellulitis, and was taken to the operating room for wound debridement.

**Figure 1:**
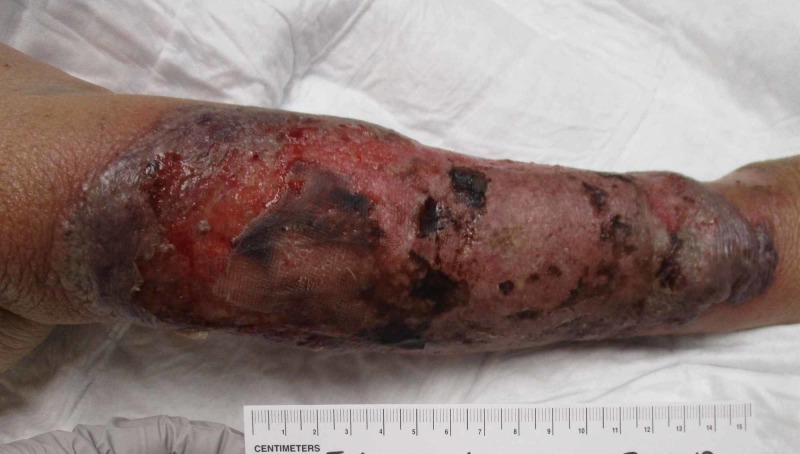
Wound at initial presentation (day 0), measuring 19.7 x 13 x 0.1 cm.

At her initial visit at the wound care clinic (one week after the date of injury), the patient reported pain and serosanguinous drainage associated with the wound. On examination, the lesion was 19.7 x 13 cm with indurated borders, a blood-filled bulla, and a shallow crater within the wound with no periwound erythema (Figure 1). She was advised to return to the hospital for an urgent hematological evaluation to exclude the possibility of warfarin toxicity. She was again started on IV antibiotics for suspected cellulitis. 

**Figure 2:**
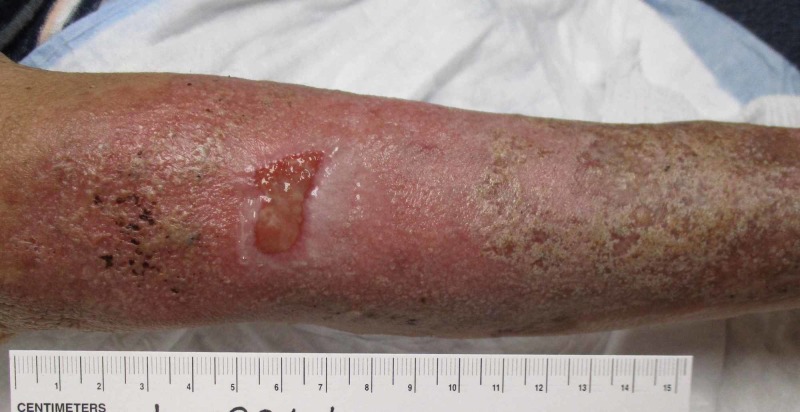
Wound at day 14. One week after initiation of steroid therapy.

**Figure 3:**
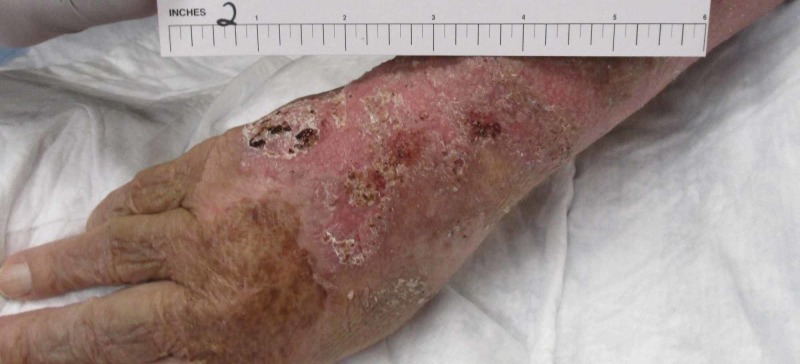
Wound at day 21, status post two weeks of steroid therapy.

**Figure 4:**
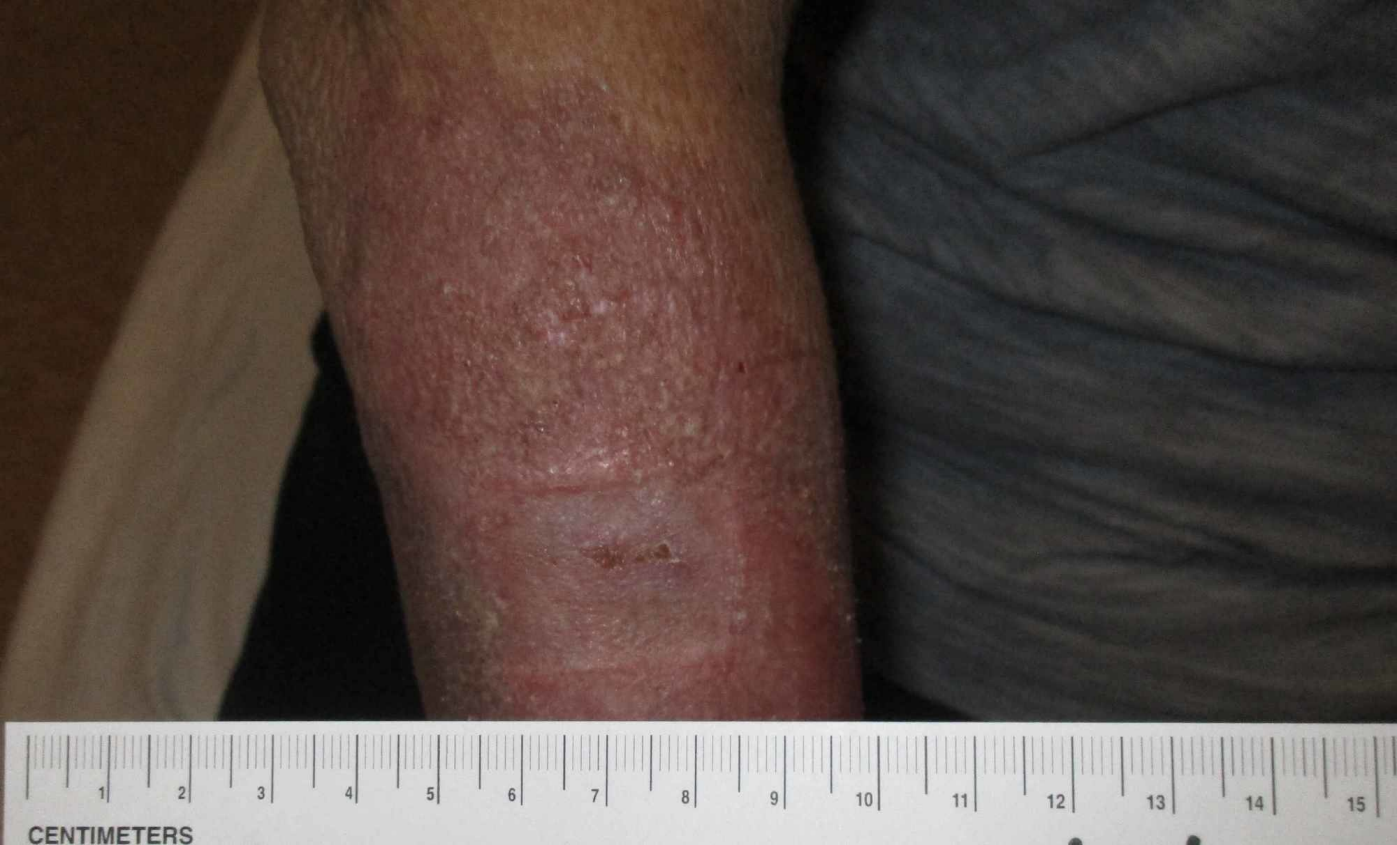
Wound at day 28, status post three weeks of steroid therapy.

A week later, the patient returned to the wound care clinic for follow-up demonstrating a persistently swollen and tender wound. Labs from the hospital were reviewed at this point which revealed the presence of lupus anticoagulant, antinuclear antibodies at a titration > 1:640 (mixed pattern), phosphatidylserine/prothrombin antibodies, cardiolipin antibodies, and beta-2 glycoprotein antibodies. A diagnosis of generalized ACLE was made, and the patient was started on oral steroids with dramatic improvement in the wound (Figures 2 and 3). Three weeks after the initiation of treatment, the only evidence that remained of the wound was dyspigmentation (Figure 4).

## Discussion

In this case, the diagnosis of ACLE was delayed due to the unique presentation and the necessity to rule out more acute and life-threatening diagnoses, such as cellulitis and warfarin-induced skin necrosis. Despite being leukopenic and afebrile with sterile wound cultures throughout both of her hospital admissions, the patient was diagnosed and treated for cellulitis. Additionally, during the second admission, she was worked up for warfarin-induced skin necrosis, which was excluded on the timing of the wound’s development and its location. Warfarin toxicity typically occurs soon after the initiation of warfarin and affects areas of adiposity, whereas the patient presented in this case had been on warfarin for many years and the wound did not involve an area of adiposity [5]. 

Once the diagnosis of ACLE is made, patients are typically started on antimalarials, including hydroxychloroquine, chloroquine, and quinacrine, as they have been the gold standard for treatment of CLE for decades. However, antimalarials can take two to three months to reach maximum efficacy, thus it may be necessary to use a bridging therapy, such as systemic corticosteroids. Because the patient presented with an advanced form of the disease, it was important to begin therapy with oral prednisone as soon as possible, as it has been shown to be beneficial in the treatment of severe cutaneous lupus and is rapidly effective. After the initiation of steroid therapy, the patient followed up with a rheumatologist who started her on hydroxychloroquine. Further systemic treatment options for CLE include methotrexate, rituximab, retinoids, dapsone, thalidomide, mycophenolate mofetil, and azathioprine. In addition to the pharmacological treatment options for ACLE, it is crucial to educate the patient on the importance of sun protection and tobacco cessation [1-3,6,7].

Although not done in this case, an important next step would be to obtain a biopsy of the lesion and perform light microscopy in order to provide histological evidence to support the clinical diagnosis made. According to the American College of Rheumatology and European League Against Rheumatism, even prior to the onset of the skin lesion, this patient did meet the criteria for a diagnosis of SLE due to her thrombocytopenia, history of seizure disorder, and presence of antiphospholipid antibodies [8]. Thus, this was presumed to be a cutaneous manifestation of the disease. Further labs to consider, in addition to a skin biopsy, include anti-dsDNA and anti-Sm antibodies, as these are highly specific for the diagnosis of SLE. 

## Conclusions

This case report demonstrates the importance of including ACLE in the differential of a patient that presents with a lesion resembling cellulitis that fails antibiotic therapy and wound debridement. Furthermore, it is imperative to establish a diagnosis of SLE as early as possible when a patient meets the criteria of the disease in order to point future providers of that patient in the right direction when a cutaneous manifestation arises. If an earlier diagnosis of SLE was made, this patient could have potentially avoided surgical debridement, multiple IV antibiotics, and two prolonged hospitalizations. Although this patient experienced a series of unfortunate events, she became eligible for kidney transplantation due to the discovery of SLE and is currently being evaluated for it.
